# Down-regulation of long non-coding RNA RP11-708H21.4 is associated with poor prognosis for colorectal cancer and promotes tumorigenesis through regulating AKT/mTOR pathway

**DOI:** 10.18632/oncotarget.15846

**Published:** 2017-03-02

**Authors:** Longci Sun, Chunhui Jiang, Chunjie Xu, Hanbing Xue, Hong Zhou, Lei Gu, Ye Liu, Qing Xu

**Affiliations:** ^1^ Department of Gastrointestinal Surgery, Renji Hospital, School of Medicine, Shanghai Jiao Tong University, Shanghai 200127, China; ^2^ Division of Gastroenterology and Hepatology, Key Laboratory of Gastroenterology and Hepatology, Ministry of Health, Renji Hospital, School of Medicine, Shanghai Jiao Tong University, Shanghai Institute of Digestive Disease, Shanghai 200001, China

**Keywords:** colorectal cancer, long noncoding RNA, RP11-708H21.4, RNA sequencing, tumorigenesis

## Abstract

Long non-coding RNAs (lncRNAs) serve critical roles in cancer development and progression. Herein, through next generation RNA sequencing and experimental validations, we determined the expression status of RP11-708H21.4 in colorectal cancer (CRC) and explored its clinical significance and biological functions in CRC. Differentially expressed lncRNAs from CRC samples and corresponding normal mucosa tissues was screened through RNA sequencing, and RP11-708H21.4 was selected for further experimental validation. The expression levels of RP11-708H21.4 in CRC tissues and cell lines were determined using qRT-PCR. Also, the relationship between the clinicopathological features and RP11-708H21.4 expression was analyzed. Cell viability was examined by CCK-8 and colony assays; cell migration and invasion were detected by transwell assays; cell cycle and cell apoptosis were analyzed by flow cytometry. The chemosensitivity of CRC cells to 5-Fluorouracil (5-FU) was also determined using CCK-8 assay. CRC xenograft tumor models were established to determine the biological functions of RP11-708H21.4 *in vivo*. Levels of cell cycle-related proteins and AKT/mTOR pathway-related proteins were detected by western blot assay. RP11-708H21.4 expression was aberrantly decreased in CRC, and its expression was closely associated with aggressive clinicopathologic features and unfavorable prognosis of CRC patients. Overexpressed RP11-708H21.4 suppresses CRC cell proliferation through inducing G1 arrest. Moreover, up-regulation of RP11-708H21.4 inhibits cell migration and invasion, causes cell apoptosis, and enhances 5-FU sensitivity of CRC cells. Finally, increased RP11-708H21.4 expression blocked AKT/mTOR pathway, and repressed *in vivo* CRC xenograft tumor growth. The results indicated that RP11-708H21.4 might have potential roles as a biomarker and a therapeutic target for CRC.

## INTRODUCTION

The colon/rectum (colorectum) is one of the most prevailing tumor sites, and colorectal cancer (CRC) ranks the third most frequent cancer and the fourth most leading reason of cancer-associated mortality globally, with approximately 1.2 million new cases and 0.6 million deaths reported each year [1, 2]. CRC is becoming more prevalent in developing countries, especially in China [3], and its occurrence rate has an elevating trend with the development of economy and living standard [4]. Although encouraging progress in diagnosis and cancer therapeutic methods has been made during the past several years, the overall survival rates of CRC patients still remain unfavorable [2]. The development and progression of CRC are often caused by the aberrant accumulation of genetic and epigenetic alternations [5]. However, the underlying molecular mechanisms which modulate the metastasis and recurrence in CRC still remain largely unknown. Accordingly, it is essential to identify some novel molecular markers to raise the efficiency of tumor diagnosis and to predict prognosis of CRC patients or even for therapeutic application.

CRC carcinogenesis is closely correlated to various alterations in oncogenes and tumor suppressor genes. Currently, mounting data from whole genome and transcriptome studies have suggested that approximately 98% of the human genome could be transcripted into short or long non-coding RNA [6, 7]. Among them, the long non-coding RNAs (lncRNAs) are a family of transcripts ranging in length from 200 nt to 100 kb with limited protein-coding capacity [8]. Most of the currently known lncRNAs exert their functions through participating in regulation of diverse cellular processes at the epigenetic, transcriptional and post-transcriptional levels [9]. LncRNAs are usually expressed in a spatial- and temporal-specific manner, making these molecules attractive therapeutic targets and pointing toward specific functions for lncRNAs in development and diseases, such as human cancer [10, 11]. For example, HOTAIR, a well-characterized lncRNA, aberrantly expressed in multiple tumors, could facilitate metastasis via regulating the chromatin state [12, 13]. More and more evidence have revealed the critical role of lncRNAs as having oncogenic and tumors suppressor functions in tumorigenesis.

Recently, a great number of lncRNAs have been identified to participate in the development and progression of CRC, including 91H [14], ZFAS1 [15], HULC [16] and CASC11 [17]. In the current study, through screening the lncRNA expression from three pairs of primary CRC samples and corresponding normal mucosa tissues using bioinformatics approaches, we determined a novel lncRNA that displayed a down-regulated expression level in CRC tissues in comparison to corresponding noncancerous tissues, which was selected for further functional analysis.

## RESULTS

### Differentially expressed lncRNAs in CRC tissues

**Figure 1:**
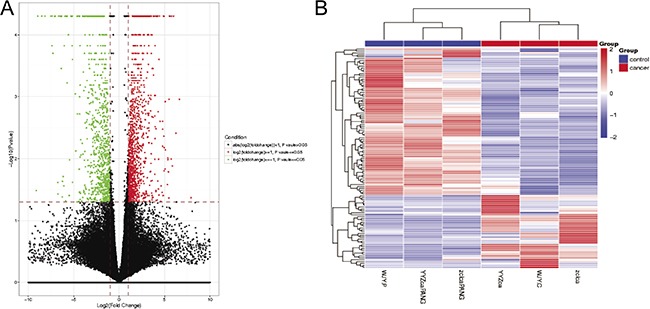
(**A**) Volcano plot of P values as a function of the weighted fold change for lncRNAs in CRC tissues. (**B**) Heatmap of expression profiles for the 117 lncRNAs that showed significant expression changes (39 up-regulated and 78 down-regulated).

Fragments per kilobase of exon per million mapped fragments (FPKMs) were calculated for normalization of the expression pattern of each lncRNA. With *P* < 0.05 and |log_2_FC|≥1.0 as the threshold, a total of 39 up-regulated and 78 down-regulated lncRNAs were identified in primary CRC samples compared with adjacent normal mucosa tissues using Student's *t*-test method, as depicted in the volcano plot (Figure 1A) and the heat map (Figure 1B). Among them, one up-regulated lncRNA, CCAT1 [18], was the known oncogene, while three down-regulated lncRNAs, including LINC01133 [19], LINC00261 [20] and RP11-789C1.1 [21], were the known tumor suppressors. Top 10 up- and down-regulated lncRNAs in CRC tissues were detailedly listed in Table 1. Here, we selected a novel lncRNA, RP11-708H21.4, for subsequent analysis.

**Table 1:** Top 10 up- and down-regulated lncRNAs in CRC tissues

Gene ID	Gene Name	log2 FC	*P* value	Regulation
ENSG00000262973	RP11-708H21.4	−4.62304	0.00005	Down
XLOC_076061	−	−4.241	0.00495	Down
ENSG00000259459	RP11-321G12.1	−4.22	0.00875	Down
XLOC_039763	−	−3.77788	0.00765	Down
XLOC_066453	−	−3.50872	0.00185	Down
ENSG00000261441	RP11-217B1.2	−3.41861	0.0462	Down
ENSG00000249096	RP11-290F5.1	−3.33067	0.03675	Down
ENSG00000232855	AF131217.1	−3.24414	0.0013	Down
ENSG00000235545	RP11-230B22.1	−3.23224	0.04335	Down
ENSG00000224189	HAGLR	−3.08186	0.00065	Down
ENSG00000250829	RP11-11N5.1	5.49651	0.0011	Up
ENSG00000272218	RP11-11N5.3	5.37744	0.0104	Up
ENSG00000260604	RP1-140K8.5	5.05226	0.0147	Up
XLOC_027973	−	4.95957	0.00665	Up
XLOC_020947	−	4.9294	0.00435	Up
ENSG00000270066	SCARNA2	4.046194	0.03395	Up
ENSG00000254560	BBOX1-AS1	4.04075	0.0328	Up
ENSG00000213468	FIRRE	3.84082	0.00005	Up
ENSG00000232445	RP11-132A1.4	3.63084	0.00035	Up
XLOC_075555	−	3.58493	0.03605	Up

### RP11-708H21.4 was significantly decreased in CRC tissues and cell lines

**Figure 2:**
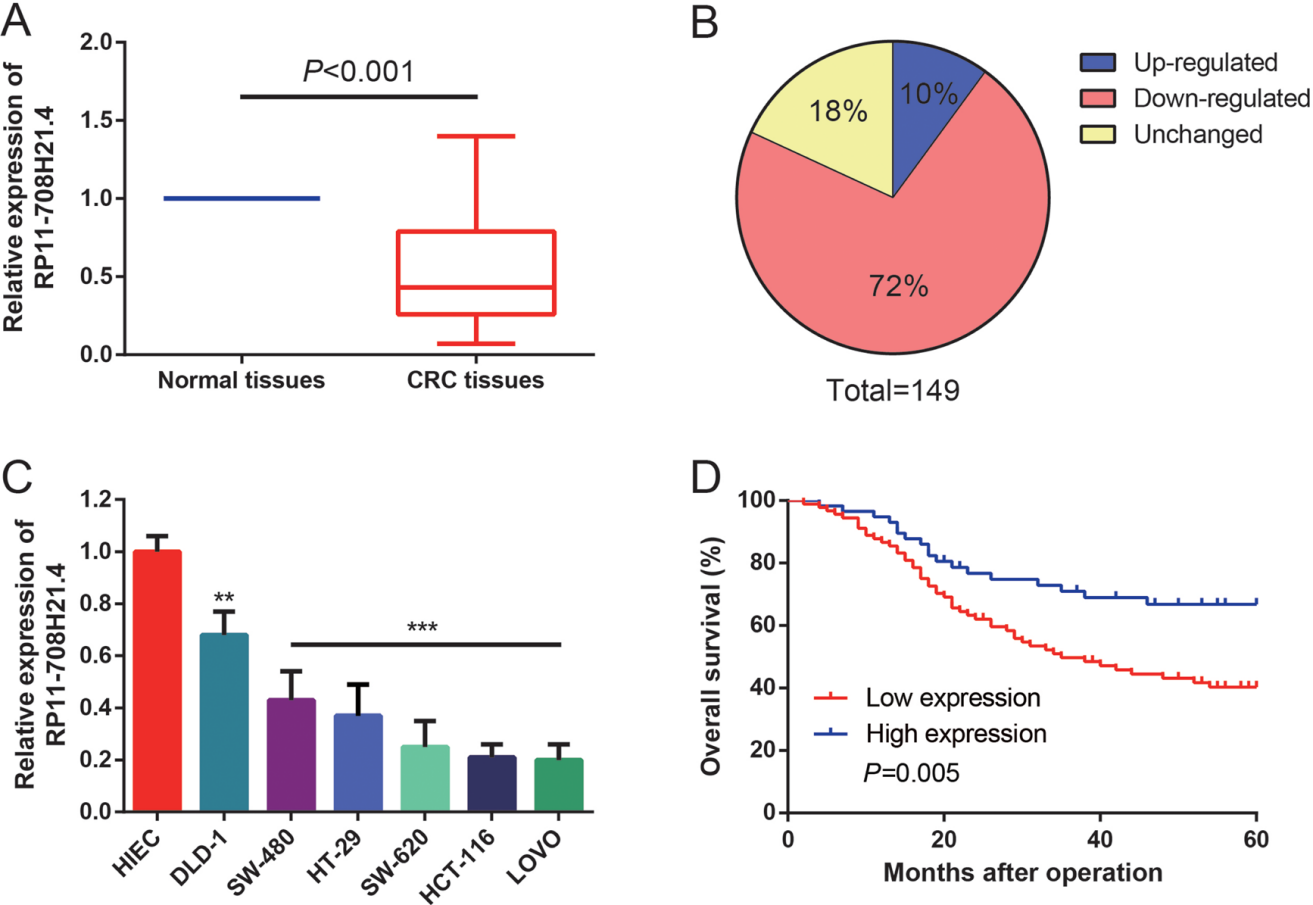
RP11-708H21.4 was significantly down-regulated in CRC (**A**) Levels of RP11-708H21.4 were determined in 149 CRC tissues and their corresponding normal mucosa by qRT-PCR. (**B**) RP11-708H21.4 was evidently down-regulated in 72% of CRC tissues compared with their corresponding normal mucosa. (**C**) Levels of RP11-708H21.4 were determined in six CRC cell lines (DLD-1, SW-480, HT-29, SW-620, HCT-116 and LOVO) and normal intestinal epithelial cell line (HIEC). Data are presented as means ± SDs based on at least three independent experiments. Asterisk indicates statistically significant changes: ***P < 0.001; **P < 0.01 compared with NC; Student's *t*-test. (**D**) Kaplan-Meier survival curve analysis showed that lower expression of RP11-708H21.4 is correlated with shorter overall survival of CRC patients. *P* value was assessed by log-rank test.

To investigate whether RP11-708H21.4 was involved in the tumorigenesis of CRC, we firstly detected the expression of RP11-708H21.4 in CRC tissues and cell lines. As shown in Figure 2A–2B, in an expression cohort of 149 CRC patients, RP11-708H21.4 levels were markedly reduced in nearly 72% of CRC tissues (107/149) compared to the corresponding non-tumor tissues (*P* < 0.05).

The levels of RP11-708H21.4 were further assessed through qRT-PCR in six CRC cell lines (DLD-1, SW-480, HT-29, SW-620, HCT-116 and LOVO) and normal human intestinal epithelial cell line (HIEC). As shown in Figure 2C, remarkable down-regulation of RP11-708H21.4 could also be found in six CRC cell lines than that of HIEC cell line (all *P* < 0.01). Taken together, these results indicated that RP11-708H21.4 might serve a critical role in CRC progression.

### Low expression of RP11-708H21.4 is correlated with poor prognosis of CRC patients

We divided the 149 CRC patients into a high RP11-708H21.4 expression group (above the average RP11-708H21.4 expression, *n* = 58) and a low expression group (below the average RP11-708H21.4 expression, *n* = 91). As presented in Table 2, there was no obvious difference between age, gender, smoking status, drinking status or tumor site and RP11-708H21.4 expression (all *P* > 0.05). However, RP11-708H21.4 expression was closely associated with tumor size (*P* = 0.026), differentiation (*P* = 0.003), TNM stage (*P* < 0.001) and lymph node metastasis (*P* = 0.033).

**Table 2:** Relationship between RP11-708H21.4 expression and clinicopathological characteristics of CRC patients (n = 149)

Characteristics	Total number	RP11-708H21.4 expression		*P* value
		Low (*n* = 91)	High (*n* = 58)	
Age				> 0.05
< 50	71	42	29	
≥ 50	78	49	29	
Gender				> 0.05
Male	89	57	32	
Female	60	34	26	
Smoking status				> 0.05
Yes	31	19	12	
No	118	72	46	
Drinking status				> 0.05
Yes	26	15	11	
No	123	76	47	
Tumor site				> 0.05
Colon	63	39	24	
Rectum	86	52	34	
Tumor size (cm)				0.026
< 5	94	51	43	
≥ 5	55	40	15	
Differentiation				0.003
Well, moderate	80	40	40	
Poor	69	51	18	
TNM stage				< 0.001
Stage I/II	75	35	40	
Stage III/IV	74	56	18	
Lymph node metastasis				0.033
Absent	61	31	30	
Present	88	60	28	

TNM, tumor-node-metastasis staging system.

Survival analyses were conducted to assess the association between RP11-708H21.4 expression and prognosis of 149 CRC patients by Kaplan-Meier survival curves and log-rank test. The results showed that the expression levels of RP11-708H21.4 were significantly associated with OS (*P* = 0.005, Figure 2D).

### RP11-708H21.4 suppresses CRC cell proliferation through induction of cell cycle arrest

To further assess the biological function of RP11-708H21.4 in CRC, we first examined the impact of RP11-708H21.4 expression on the *in vitro* proliferation of CRC cells. Overexpression of RP11-708H21.4 was achieved through transient transfection of pcDNA3.1-RP11-708H21.4 into HT-29 and HCT-116 cell lines. Following transfection of pcDNA3.1-RP11-708H21.4, RP11-708H21.4 expression was significantly increased as compared to the same cells transfected with empty vector (Data not shown).

**Figure 3:**
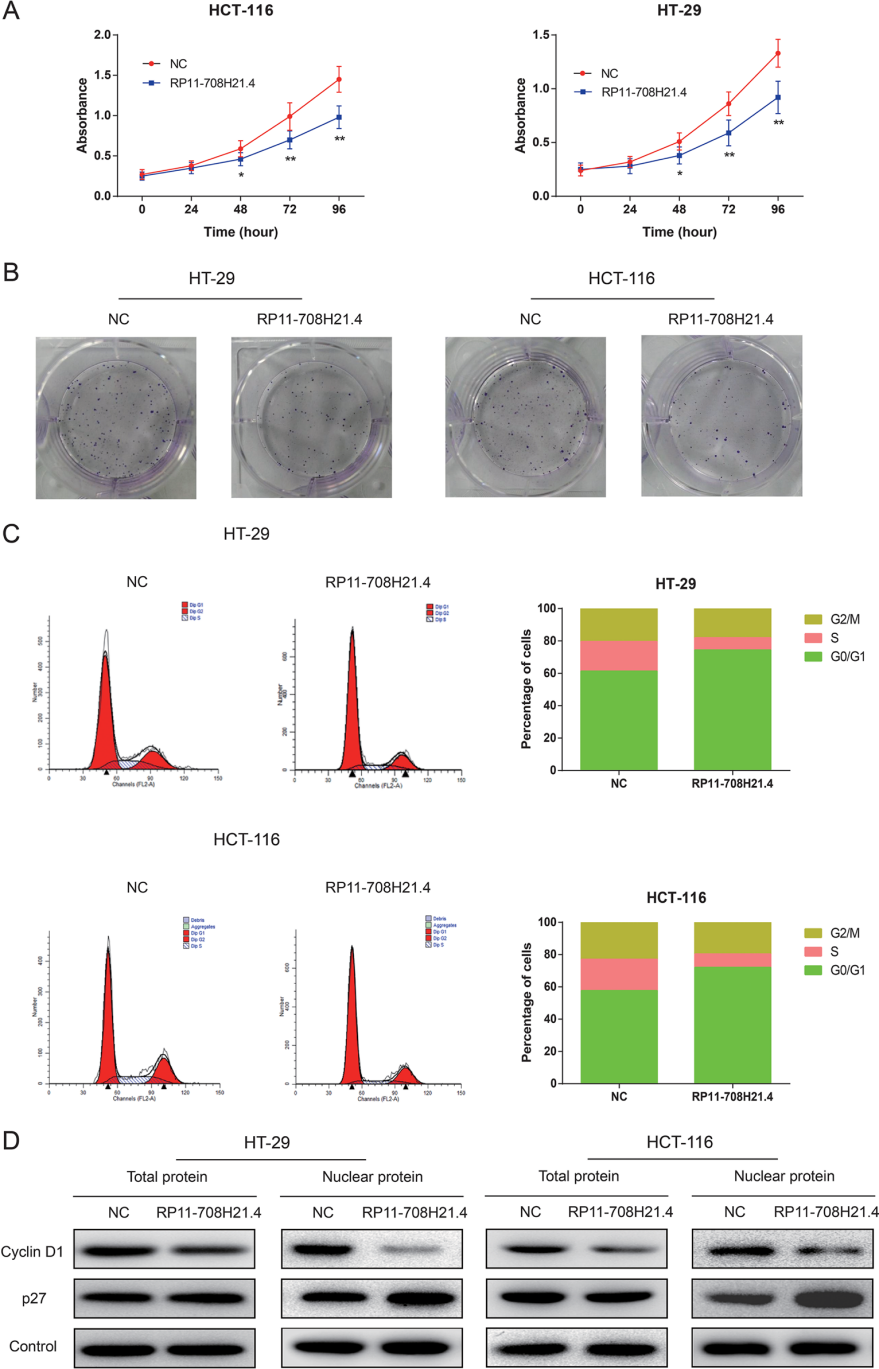
RP11-708H21.4 suppresses CRC cell proliferation *in vitro* through induction of cell cycle arrest (**A**) CCK-8 assay was performed to measure the proliferation of HT-29 and HCT-116 cell lines transfected with empty vector (control) or pcDNA3.1-RP11-708H21.4. (**B**) The representative pictures of colony formation assay for crystal violet-stained HT-29 and HCT-116 cells were shown. (**C**) Cell cycle analysis showed that significant G1 phase arrest and S phase decrease were observed in HT-29 and HCT-116 cell lines transfected with empty vector (control) or pcDNA3.1-RP11-708H21.4. (**D**) Western blotting showed that Cyclin D1 expression was decreased, whereas p27 expression was increased at nuclear protein levels after the up-regulation of RP11-708H21.4 in HT-29 and HCT-116 cells. Data are presented as means ± SDs based on at least three independent experiments. Asterisk indicates statistically significant changes: **P < 0.01; *P < 0.05 compared with NC; Student's *t*-test.

Growth curves determined by CCK8 assay revealed that, after overexpression of RP11-708H21.4, the proliferation ability of HT-29 and HCT-116 cells was markedly repressed (all *P* < 0.01, Figure 3A). In addition, the colony formation assay also demonstrated that the colony formation rates of RP11-708H21.4 overexpressed HT-29 and HCT-116 cells were much lower than those transfected with empty vector, as shown in Figure 3B. These findings indicated that RP11-708H21.4 might exert the potential to suppress the proliferation of CRC cells.

Dysregulation of cell cycle transition is a hallmark of cancer cell [22]. Thus, cell cycle progression was further analyzed to investigate the influence of RP11-708H21.4 on CRC cell proliferation. As demonstrated in Figure 3C, compared with the negative control, RP11-708H21.4 overexpression caused a dramatic decrease in HT-29 and HCT-116 cells in S-phase and a remarkable accumulation of HT-29 and HCT-116 cells at G0/G1-phase. Western blotting showed that p27 expression was greatly increased, whereas Cyclin D1 expression was greatly decreased at nuclear protein levels after the overexpression of RP11-708H21.4 in HT-29 and HCT-116 cells (Figure 3D).

### RP11-708H21.4 inhibits CRC cell invasion, and causes cell apoptosis

**Figure 4:**
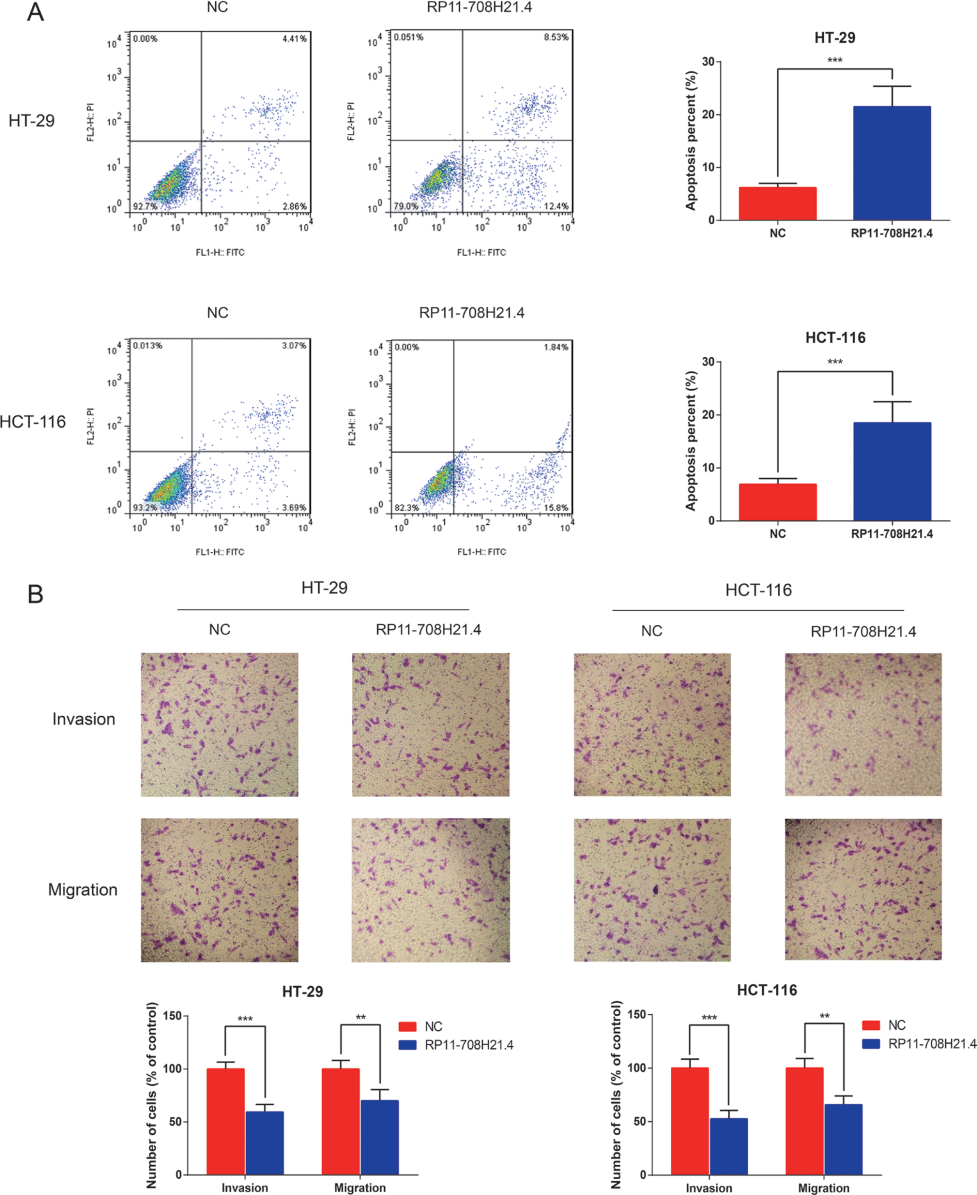
RP11-708H21.4 facilitates CRC cell apoptosis and represses cell invasion *in vitro* (**A**) Cell apoptosis analysis of HT-29 and HCT-116 cell lines transfected with empty vector (control) or pcDNA3.1-RP11-708H21.4. (**B**) Transwell assay was performed to measure the invasion and migration of HT-29 and HCT-116 cell lines transfected with empty vector (control) or pcDNA3.1-RP11-708H21.4. The cells were counted under a microscope in five randomly selected fields. Data are presented as means ± SDs based on at least three independent experiments. Asterisk indicates statistically significant changes: ***P < 0.001; **P < 0.01 compared with NC; Student's *t*-test.

To investigate the potential mechanisms underlying growth suppression after RP11-708H21.4 overexpression, we assessed its effect on cell apoptosis in CRC cells. The results demonstrated that RP11-708H21.4 overexpression significantly promoted apoptosis in HT-29 and HCT-116 cells (all *P* < 0.001, Figure 4A).

Since metastasis of cancer cells is considered as a critical aspect of cancer progression, we detected the effect of RP11-708H21.4 on migration and invasion capacities of CRC cells via performing transwells assays. The results demonstrated that overexpressed RP11-708H21.4 evidently repressed the migration and invasion capacities of HT-29 and HCT-116 cells (all *P* < 0.01, Figure 4B).

### RP11-708H21.4 enhances 5-FU sensitivity in CRC cells

**Figure 5:**
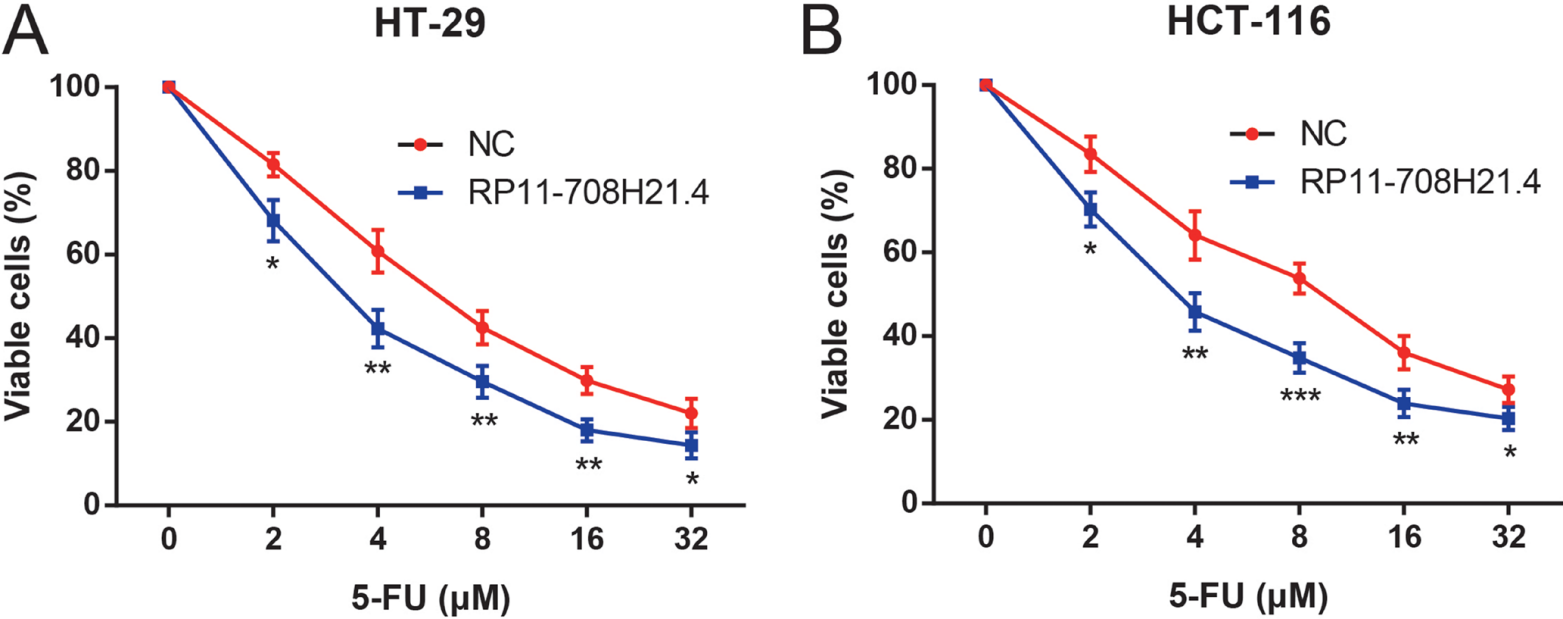
RP11-708H21.4 enhances 5-FU sensitivity in CRC cells CCK-8 assay was conducted to detect the growth of (**A**) HT-29 and (**B**) HCT-116 cell lines transfected with empty vector (control) or pcDNA3.1-RP11-708H21.4 at different concentrations of 5-FU (2, 4, 8, 16 and 32 μM). Data are presented as means ± SDs based on at least three independent experiments. Asterisk indicates statistically significant changes: ***P < 0.001; **P < 0.01; *P < 0.05 compared with NC; Student's *t*-test.

To explore the role of RP11-708H21.4 as a tumor suppressor, we investigated the RP11-708H21.4-regulated chemosensitivity of CRC cells. Since 5-FU is a first-line chemotherapeutic agent for treatment of CRC patients, the sensitivities of HT-29 and HCT-116 cells to 5-FU with or without overexpression of RP11-708H21.4 were thus assessed. As demonstrated in Figure 5, 5-FU exerted a significant inhibitory effect on the cell viabilities of CRC cells in a dose-dependent manner. In addition, HT-29 and HCT-116 cells transfected with pcDNA3.1-RP11-708H21.4 exhibited more impaired cell viabilities under 5-FU treatments compared with those transfected with empty vector. Taken together, our results suggest overexpression of RP11-708H21.4 could effectively sensitize CRC cells to chemotherapeutic agents.

### RP11-708H21.4 blocks AKT/mTOR pathway in CRC cells

**Figure 6:**
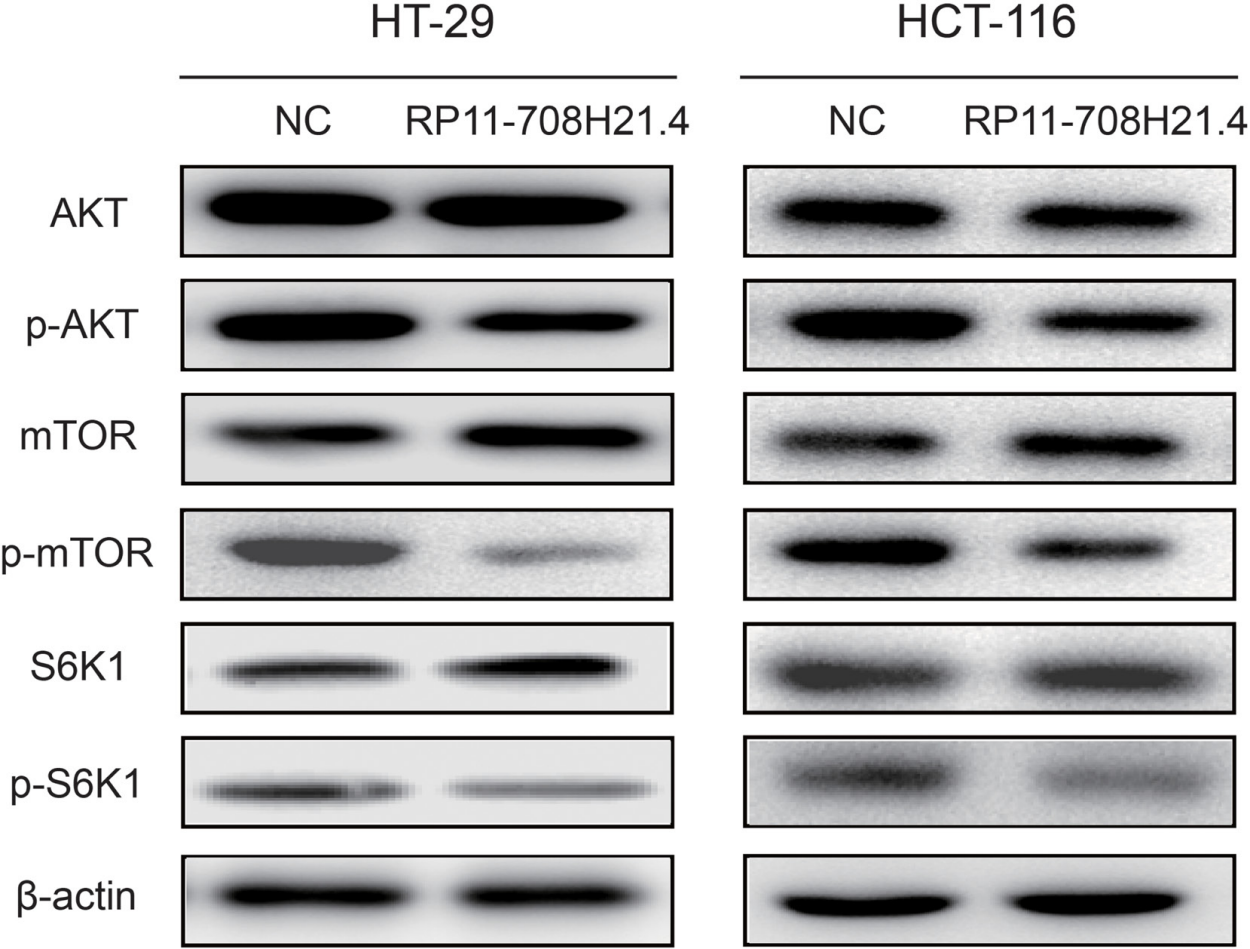
RP11-708H21.4 blocks AKT/mTOR pathway in CRC cells Western blotting showed that phosphorylation of AKT, mTOR and S6K1 was significantly repressed in RP11-708H21.4 overexpressed HT-29 and HCT-116 cells.

AKT/mTOR pathway plays important roles in regulating cell cycle and cell apoptosis. To explore whether the effects of RP11-708H21.4 on CRC cells is associated with this pathway, we assessed the activity of RP11-708H21.4 on the representative signal proteins in the pathway. As demonstrated in Figure 6, phosphorylation of AKT, mTOR, as well as S6K1, was significantly suppressed after transfection of pcDNA3.1-RP11-708H21.4 in HT-29 and HCT-116 cells. These results suggested that RP11-708H21.4 blocks AKT/mTOR pathway in CRC cells.

### RP11-708H21.4 represses CRC xenograft tumor growth *in vivo*

**Figure 7:**
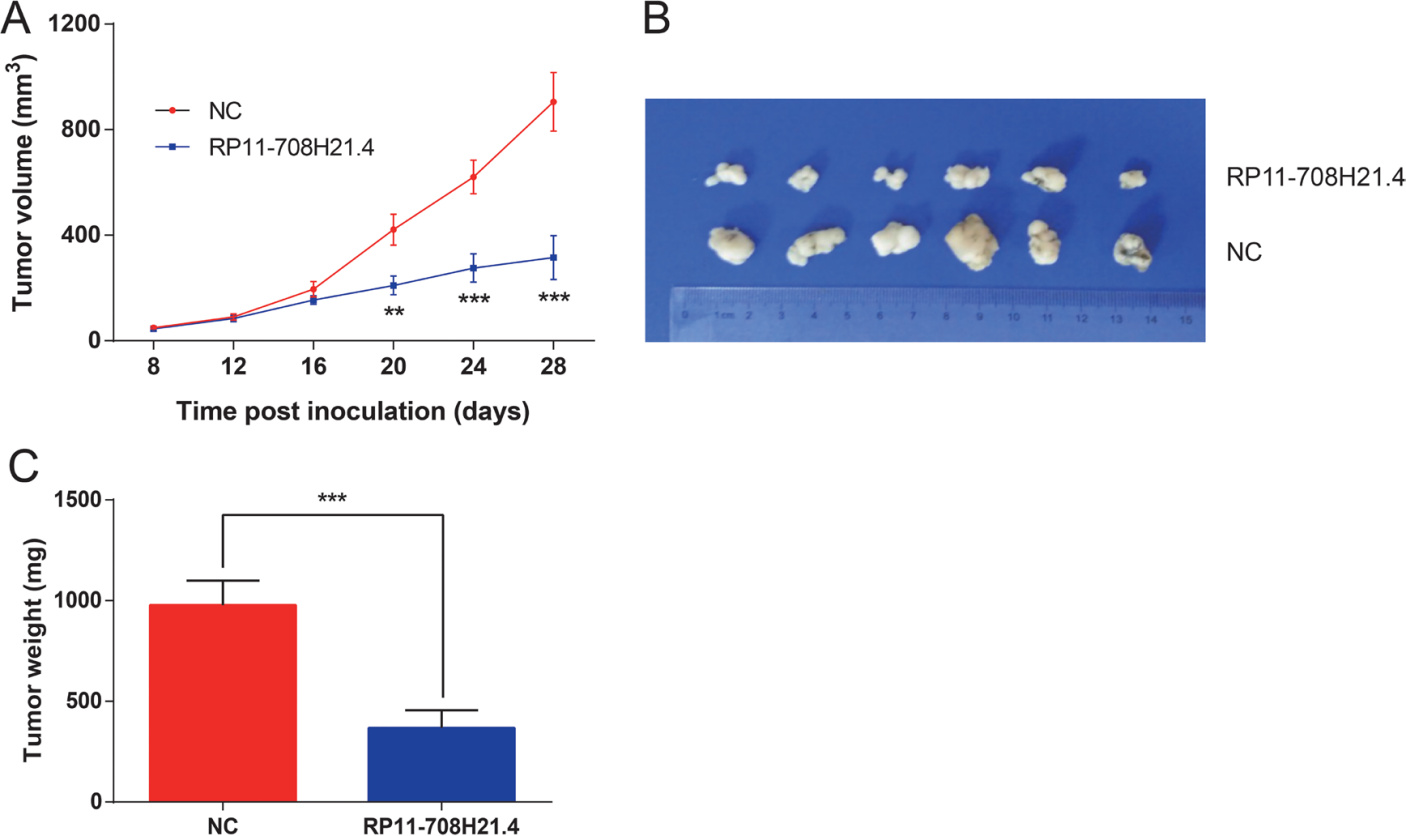
RP11-708H21.4 represses CRC xenograft tumor growth *in vivo* (**A**) CRC xenograft tumor models were developed in nude mice by HT-29 cells transfected with empty vector (control) or pcDNA3.1-RP11-708H21.4. Tumor volume was calculated every 3 days. (**B**) The representative pictures of tumors from respective groups of nude mice were shown. (**C**) Tumor weight of each tumor sample from two groups was represented. Data are presented as means ± SDs based on at least three independent experiments. Asterisk indicates statistically significant changes: ***P < 0.001; **P < 0.01 compared with NC; Student's *t*-test.

Next, we investigated whether high levels of RP11-708H21.4 could suppress tumor growth *in vivo*. HT-29 cells transfected with pcDNA3.1-RP11-708H21.4 or empty vector were inoculated into the nude mice. As shown in Figure 7A, the tumors in the pcDNA3.1-RP11-708H21.4 group grew more slowly than those in the empty vector group. Four weeks after injection, tumors dissected from the mice of pcDNA3.1-RP11-708H21.4 group were markedly smaller than the control tumors (Figure 7B). Moreover, the tumor weight was significantly lower in the pcDNA3.1-RP11-708H21.4 group compared with the empty vector group (*P* < 0.001, Figure 7C), indicating RP11-708H21.4 represses CRC xenograft tumor growth *in vivo*.

## DISCUSSION

With a 5-year survival rate lower than 10% [23], there is a clear and present need for improvement of the clinical care of CRC patients. Despite great advances in our understanding of the molecular mechanisms of CRC, at present the prognosis for CRC patients remains unsatisfactory. Tumor progression in CRC is a multi-step process involving a large number of genetic and epigenetic alterations [24]. To date, it has been estimated that approximately 15,000 lncRNAs are present in the human genome [25]. A growing amount of research has documented that lncRNAs are featured as crucial regulators during CRC tumorigenesis, contributing to tumor proliferation, apoptosis, and survival [26]. For example, Wan and colleagues reported that HOTAIRM1 expression was increased in CRC tissues, and up-regulated levels of HOTAIRM1 might function as a promising prognostic indicator for CRC patients [27]. Therefore, there is an ongoing effort to elucidate novel dysregulated lncRNAs and their possible roles in CRC progression.

Substantial advances in next-generation sequencing technology (RNA-Seq) have revolutionized omics and biomedical sciences, particularly in the domain of cancer research [28]. RNA-seq data are expected to be less noisy in comparison to microarray-based expression profiling [29, 30], and therefore, they provide a more accurate and comprehensive understanding of cancer progression at the molecular level. In this study, lncRNA profiling by RNA-seq was used to successfully detect a number of statistically significant novel lncRNAs related to CRC, and for the first time identify RP11-708H21.4 as a critical onco-suppressive lncRNA in CRC. We found that compared with corresponding noncancerous tissues, the average level of RP11-708H21.4 in human CRC tissues was remarkably decreased. Moreover, the lower expression of RP11-708H21.4 was closely correlated to the aggressive clinicopathologic features and unfavorable prognosis of CRC patients.

The importance of lncRNAs in human cancer might be correlated with their abilities to impact cellular functions. To further determine the biological functions of RP11-708H21.4 in CRC, we determined the influence of gain of function of RP11-708H21.4 on CRC cell growth and invasion. Our observations suggested that overexpression of RP11-708H21.4 suppresses *in vitro* cell proliferation, migration, and invasion, and caused cell cycle arrest at the G0/G1-phase. Also, increased RP11-708H21.4 levels remarkably suppressed the growth of CRC cells in murine model. 5-FU has been extensively used in clinical practice as the most critical chemotherapeutic drug for CRC treatment, but chemoresistance to 5-FU confines its therapeutic efficacy [31, 32]. Herein, we found that RP11-708H21.4 has an important role in enhancing 5-FU sensitivity of CRC cells, indicating RP11-708H21.4 may increase the therapeutic efficacy of 5-FU in CRC treatment.

Furthermore, we detected potential targets that could be potentially responsible for RP11-708H21.4-mediated cell cycle arrest. The present experimental results demonstrated that Cyclin D1 expression was significantly reduced and p27 expression was significantly elevated at nuclear protein levels in CRC cells with RP11-708H21.4 overexpression. Cyclin D1 is one of the most crucial cell cycle regulatory proteins, serving pivotal roles in the pathogenesis of several human tumor types including CRC [33, 34]. Cyclin D1 assembles with its partners such as cyclin-dependent kinase (CDK) 4 and CDK6, which subsequently enters the nucleus and phosphorylates tumor suppressor protein retinoblastoma (Rb), leading to progression from the G1 to the S phase [35]. By contrary, CDK inhibitors, including p27, are found to block the cell cycle transition through repressing several Cyclin-CDK complexes [36]. Thus, we speculated that Cyclin D1 and p27 might be the downstream regulators involved in RP11-708H21.4-mediated growth arrest in CRC. However, the precise mechanisms by which RP11-708H21.4 regulates Cyclin D1 and p27 expression remains elusive and requires further detection.

A previous study showed that inactivation of mTOR signaling could inhibit Cyclin D1 expression, mainly due to the enhanced instability of Cyclin D1 mRNA and protein [37], thereby repressing cell proliferation. The AKT/mTOR signaling is a pivotal pathway participating in multiple physiological and pathological processes, including gene transcription, protein translation, cell cycle regulation and proliferation [38]. AKT serves as a pivotal regulator of both protein synthesis and degradation through modulating the activity of the mTOR pathway [39]. Blockade of AKT/mTOR signaling pathway has been considered to be useful in cancer treatment [40, 41]. Based on our present findings, we considered RP11-708H21.4 might regulate Cyclin D1 and p27 expression partly through inactivation of AKT/mTOR pathway. Further investigations are required to gain insight into the mechanisms by which RP11-708H21.4 regulates AKT/mTOR signaling pathway.

In conclusion, the RNA-Seq data and analyses enabled us to for the first time find that RP11-708H21.4, an lncRNA which located in the 17q21 gene desert region, exerts onco-suppressive functions in the development and progression of CRC, and might potentially act as a novel and powerful therapeutic candidate and diagnostic predictor for future CRC treatment. However, all tissue samples in the present article were only collected from a single hospital, and the sample size was limited. A larger, prospective, randomized and multicenter study should be performed to validate the correlations of RP11-708H21.4 expression with CRC in the near future.

## MATERIALS AND METHODS

### Study population and tissue collection

Primary CRC samples and paired adjacent non-tumor mucosa tissues (at least 5 cm from the margin of the tumor) were obtained from 149 patients with histologically confirmed CRC (89 male and 60 female), who received surgical resections at Department of Gastrointestinal Surgery, Renji Hospital, School of Medicine, Shanghai Jiao Tong University (Shanghai, China) between January 2009 and January 2011. Patient age ranged from 34 to 85 years (median age: 52.1 years). The diagnosis of CRC was confirmed by two experienced pathologists. Only CRC patients that had not received any preoperative neoadjuvant or adjuvant treatment were collected. The patients’ clinicopathological characteristics, obtained from medical archives and individual interviews, were recorded in Table 2. All tissues were snap-frozen immediately in liquid nitrogen after resection and stored at −80 °C until further processing. This research was approved by the Ethics Committee of Renji Hospital, and written consent was obtained from all the patients before participating in the study.

### Reagents, cell lines, and cell cultures

5-Fluorouracil (5-FU) was purchased from Sigma-Aldrich (Shanghai, China) and stored as a 20 mM solution in dimethyl sulfoxide (DMSO).

The normal human intestinal epithelial cell line (HIEC) and six human CRC cell lines (DLD-1, SW-480, HT-29, SW-620, HCT-116 and LOVO), purchased from the Cell Bank of the Chinese Academy of Sciences (Shanghai, China), were cultured in RPMI 1640 (GIBCO-BRL; Invitrogen, Carlsbad, CA, USA) and Dulbecco's modified Eagle's medium (DMEM; GIBCO-BRL; Invitrogen) respectively, each supplemented with 10% fetal bovine serum (HyClone, Logan, UT, USA) with 100U/ml penicillin and 100 mg/ml streptomycin at 37°C in a humidified air atmosphere containing 5% CO_2_.

### Sample preparation and next generation RNA sequencing

Total RNA from the frozen tissues and the cultured cells was extracted using the TRIzol reagent (Life Technologies). Agilent 2100 Bioanaylzer and ABI StepOnePlus Real-Time PCR System were used to assess the quantity and integrity of total RNA. The library was prepared using a TruSeq Stranded Total RNA Library Prep Kit (Illumina, US) and subjected to 2 × 100 base paired-end sequencing on an Illumina Hi-seq 2500 machine. Clean reads were obtained through removing dirty raw reads, reads in which the percentage of unknown bases (N) is more than 10% and low quality reads (reads with the percentage of the low quality base (base with quality value ≤ 5) greater than 50%). Clean reads from each sample were mapped to the hg19 genome sequence using the Bowtie software [42].

### Differentially expressed lncRNAs analysis

RNA-seq data were compared between three pairs of CRC tissues and matched non-tumor mucosa tissues to determine differentially expressed lncRNAs. The differentially expressed lncRNAs were identified by using the Bioconductor R package limma [43]. The *P* value was adjusted for multiple testing using the false discovery rated (FDR) method, and |log_2_ fold change (FC)|≥1.0 and adjusted *P* value < 0.05 were considered significant. Besides, hierarchical clustering analysis was conducted to exhibit the differentially expressed lncRNAs.

### Quantitative real-time PCR (qRT-PCR)

RP11-708H21.4 expression from tissue samples or the cultured cells was quantified using the FastStart Universal SYBR Green Master Mixes (Roche Diagnostics Corp., Indianapolis, IN, USA) on the ABI Prism 7500 (Applied Bio systems, Foster City, CA, USA). The relative expression levels of RP11-708H21.4 were investigated using the comparative delta-delta CT method (2^−ΔΔCt^) with human glyceraldehyde-3-phosphate dehydrogenase (GAPDH) as internal controls for normalization: ΔΔCT = (CT_RP11-708H21.4_-CT_GAPDH_)_tumor_-(CT_RP11-708H21.4_-CT_GAPDH_)_normal_ [44]. The sequences of the specific primers used in the research were as follows: GAPDH, forward: 5′-GTCAACGGATTTGGTCTGTATT-3′ and reverse: 5′-AGTCTTCTGGGTGGCAGTGAT-3′; RP11-708H21.4, forward: 5′-ACACAGGCAGAAGACTCTCC-3′ and reverse: 5′-TGCCTCTGGTCTCAAACACA-3′. All experiments were performed in triplicates.

### Plasmids and cellular transfection

The full-length RP11-708H21.4 sequence was synthesized and inserted into a pcDNA3.1 vector (Invitrogen, Shanghai, China), named pcDNA3.1-RP11-708H21.4. An empty pcDNA3.1 vector was used as a control. The vectors were prepared using DNA Midiprep kits (Qiagen, Hilden, Germany). To establish cell lines with transient overexpression of RP11-708H21.4. Cells were grown on six-well plates to 70% confluency and transfected with 10 μg pcDNA3.1-RP11-708H21.4 plasmid or control pcDNA3.1 vector using Lipofectamine 2000 reagent (Invitrogen). At 48 h post transfection, cells were harvested for further functional assays.

### Cell proliferation assay

Cell proliferation was investigated through using Cell Counting Kit-8 (CCK-8) assay kit (Beyotime, Shanghai, China). CRC cells (4000 cells per well) transfected with pcDNA3.1-RP11-708H21.4 or with negative control were allowed to grow in 96-well plates. Proliferation rates were detected at 24 hours, 48 hours, 72 hours and 96 hours after transfection. 10 μl of CCK8 solution was added to each well, and the absorbance of each well was assessed at a wavelength of 450 nm through a spectrophotometer reader. All experiments were performed in triplicates.

For the 5-FU dose-response curve, 5-FU was added to the wells at a final concentration of 2, 4, 8, 16 and 32 μM, and continued to incubate for 72 h. The absorbance of each well was thus measured.

### Colony formation assay

For colony assay, CRC cells (500 cells per well) transfected with pcDNA3.1-RP11-708H21.4 or with negative control were allowed to grow in 6-well plates. Then, the cells were cultured with medium changed every 5 days. After 10 days of incubation, cells were fixed with 4% paraformaldehyde and then stained with 0.5% crystal violet. Colony-forming rates were then analyzed. All experiments were performed in triplicates.

### Migration and invasion assay

After transfected with pcDNA3.1-RP11-708H21.4 or with negative control, a total of 3 × 10^4^ cells in 100 μl of serum-free medium were seeded into the upper chambers of an insert (8-μm pore size; Corning Costar, NY, USA) coated with or without Matrigel (Millipore, Billerica, USA). The lower chambers were filled with 600 μl of DMEM containing 10% FBS as a chemoattractant. After 24 h of incubation, the remaining cells were removed with cottons swabs, while cells on the lower membrane surface were fixed with 4% paraformaldehyde and then stained with 0.1% crystal violet. Five random fields of each chamber were photographed using a light microscope (Olympus, Tokyo, Japan). All experiments were performed in triplicates.

### Analysis of apoptosis and cell cycle by flow cytometry

For apoptosis analysis, transfected cells were double stained with FITC-Annexin V and propidium iodide (PI) using the FITC Annexin V Apoptosis Detection Kit (BD Biosciences, San Jose, CA, USA). Then, the stained cells were analyzed by a FACSCalibur flow cytometer (BD Biosciences) equipped with Cell Quest software (BD Biosciences). Cells were categorized into viable cells, early apoptotic cells, apoptotic cells and dead cells. The percentages of early and late apoptotic cells were counted and compared. For cell cycle analysis, cells were stained with PI using the CycleTEST PLUS DNA Reagent Kit (BD Biosciences) and analyzed by flow cytometry. The percentages of the cells in G0–G1, S, and G2–M phases were counted and compared. All experiments were performed in triplicates.

### Western blot assay

To detect total protein levels, cells were lysed using radioimmuno precipitation assay buffer (Solarbio, Beijing, China) containing a protein inhibitor cocktail (Sigma-Aldrich). To investigate nuclear protein levels, nucleus lysate was prepared using NE-PER Nuclear and Cytoplasmic Extraction Kit (Thermo Scientific, Rockford, IL, USA). Protein concentration was measured with the bicinchoninic acid assay (Pierce, Rockford, IL, USA). Equal amounts of protein extractions were separated by 10% SDS-polyacrylamide gels (Bio-Rad Laboratories Inc., Hercules, CA, USA), then transferred to polyvinylidene fluoride (PVDF) membranes (Millipore, Bedford, MA, USA). The members were probed with the following specific primary antibodies: Cyclin D1 (Cell Signaling Technology, Boston, MA, USA), p27 (Cell Signalling Technology), AKT (Epitomics, Burlingame, CA, USA), phosphorylated AKT (Epitomics), mTOR (Cell Signalling Technology), phosphorylated mTOR (Cell Signalling Technology), S6K1 (Cell Signaling Technology) and phosphorylated S6K1 (Abcam, Cambridge, USA), followed by incubation with horseradish peroxidase (HRP)-linked secondary antibodies (Santa Cruz Biotechnology). The membrane was rinsed, and the ECL method (ThermoScientific, Hudson, NH, USA) was used to visualize the bands. The relative densities of proteins were quantified with the Quantity One software (4.5.0 basic, Bio-Rad Laboratories), and normalized against β-actin (for total protein) or Lamin B (for nuclear protein). All experiments were performed in triplicates.

### Xenograft

All animal trials were approved by the Institutional Review Board of Renji Hospital, School of Medicine, Shanghai Jiao Tong University and were undertaken in accordance with the Institutional Animal Care and Use guidelines. Twelve four-week-old female athymic BALB/c nude mice, purchased from Shanghai Laboratory Animals Center (Shanghai, China), were housed in laminar airflow chambers under specific pathogen-free conditions. 150 μl of medium containing a total of 1 × 10^6^ CRC cells transfected with pcDNA3.1-RP11-708H21.4 or empty vector were injected subcutaneously into the flanks of each mouse (*n* = 6/group). Tumor growth was measured every 4 days using calipers, and tumor volumes were calculated according to the formula: tumor volume (mm^3^) = length × width^2^ × 0.5. The mice were sacrificed after 28 days, and the tumors were excised and weighted.

### Statistical analysis

All of the statistical analyses were performed with GraphPad Prism software (GraphPad Software Inc., La Jolla, CA, USA) and SPSS software (SPSS, Inc., Chicago, IL, USA). The statistical significance of the differences between two groups was analyzed using Student's *t*-test (for continuous data) or chi-square test (for categorical data). Kaplan-Meier survival analysis and log-rank test were performed to assess the probability of overall survival (OS), which was defined as the interval between the dates of surgery and death. Differences with a two-sided *P* value of less than 0.05 were regarded to be statistically significant.
